# Histone Deacetylase 5 in Prelimbic Prefrontal Cortex Limits Context-Associated Cocaine Seeking

**DOI:** 10.1016/j.biopsych.2025.06.027

**Published:** 2025-07-07

**Authors:** Sarah M. Barry, Jessica L. Huebschman, Derek M. Devries, Lauren M. McCue, Evgeny Tsvetkov, Rose Marie Akiki, Caroline Limbaker, Ethan M. Anderson, Daniel J. Wood, Benjamin M. Siemsen, Stefano Berto, Michael D. Scofield, Makoto Taniguchi, Rachel D. Penrod, Christopher W. Cowan

**Affiliations:** Department of Neuroscience, Medical University of South Carolina, Charleston, South Carolina (SMB, JLH, DMD, LMM, ET, RMA, CL, EMA, DJW, BMS, SB, MDS, MT, RDP, CWC)

## Abstract

**BACKGROUND:**

Repeated cocaine use produces neuroadaptations that support drug craving and relapse in substance use disorders (SUDs). Powerful associations formed with drug use environments can promote a return to active drug use in patients with SUD, but the molecular mechanisms that control the formation of these prepotent drug-context associations remain unclear.

**METHODS:**

In an animal model of intravenous cocaine self-administration (SA), we used male Sprague Dawley rats to examine the role of histone deacetylase 5 (HDAC5) in the prelimbic cortex (PrL) and infralimbic cortex in context-associated drug seeking. To this end, we employed viral molecular tools, chemogenetics, RNA sequencing, electrophysiology, and immunohistochemistry.

**RESULTS:**

In the PrL, reduction of endogenous HDAC5 augmented context-associated but not cue- or drug prime–reinstated cocaine seeking, whereas overexpression of HDAC5 in the PrL, but not in the infralimbic cortex, reduced context-associated cocaine seeking but had no effects on sucrose seeking. In contrast, PrL HDAC5 overexpression following acquisition had no effects on future cocaine seeking. We found that HDAC5 and cocaine SA altered expression of numerous PrL genes, including many synapse-associated genes. HDAC5 significantly increased inhibitory synaptic transmission onto PrL deep-layer pyramidal neurons and reduced the induction of FOS-positive neurons in the cocaine SA environment.

**CONCLUSIONS:**

Our findings reveal an essential and selective role for PrL HDAC5 to limit associations formed in cocaine, but not sucrose, SA environments, and that PrL HDAC5 alters the PrL excitatory/inhibitory balance, possibly through epigenetic regulation of synaptic genes. These results further position HDAC5 as a key factor regulating reward-circuit neuroadaptations that underlie common relapse triggers in SUDs.

Substance use disorders (SUDs) are a significant public health challenge and are associated with a reduced quality of life and increased mortality. Cocaine is the most abused stimulant-class drug, and deaths due to cocaine use have more than doubled since 2012 (1). Despite these challenges, no pharmacological agents have been approved for safe and effective treatment of cocaine use disorder (2). Repeated drug use causes transcriptional alterations in brain reward areas that support persistent, functional alterations that predispose individuals to drug seeking and relapse behavior (3,4). The medial prefrontal cortex (mPFC) is a critical component of drug-seeking circuitry due to its role in executive functions, including learning, memory retrieval, and decision making (5–10). The mPFC can be divided into subdivisions based on function and projections (11–13). Two subregions of the mPFC, the prelimbic cortex (PrL) and the infralimbic cortex (IL), undergo extensive alterations following use of illicit substances and are critical components of the circuitry involved in a return to active drug use (i.e., relapse) (14–19). The mPFC also serves as a major glutamatergic input to the nucleus accumbens (NAc), and this projection is critically involved in drug-seeking behavior (20–22).

Neuroadaptations within the mPFC, including epigenetic alterations, contribute to persistent drug-seeking (4,23,24). Posttranslational modifications to histone tails are one way that gene expression is regulated following drug experiences. Histone deacetylases (HDACs) have been shown to mediate cocaine-induced changes in cellular functions (25–29). Class IIa HDACs are unique in the HDAC family because synaptic activity, or activation of cAMP (cyclic adenosine monophosphate) signaling, can trigger a shift in their nucleocytoplasmic localization (27). One class IIa HDAC, HDAC5, is highly expressed in the mPFC (30,31), and cocaine use causes a decrease of HDAC5 messenger RNA (mRNA) in the PFC (32). In addition, HDAC5 in the NAc serves to limit both cocaine conditioned place preference (CPP) and cue- and drug prime–reinstated cocaine or heroin seeking following intravenous self-administration (IVSA) (27–29,33). Indeed, noncontingent or contingent drug exposure induces a transient nuclear accumulation of HDAC5 in NAc medium spiny neurons (MSNs), which appears to be driven in part by activation of dopamine D_1_ receptors, cAMP signaling, and dephosphorylation of 3 conserved HDAC5 serines (27). To this point, expression of a dephosphorylated, nuclear-localized HDAC5 mutant in the NAc (S259A/S279A/S498A or HDAC5-3SA) reduces the development of cocaine CPP and cued- and drug-primed cocaine seeking following SA training (28).

Despite strong cortical expression, HDAC5’s regulation and function in the mPFC, a key brain hub for drug-seeking behavior, has not been explored in SUD-related behavior. We show here that HDAC5 in the PrL, but not in the IL, plays a selective role in limiting context-associated cocaine seeking, but not sucrose seeking, and regulates numerous PrL synaptic genes, GABAergic (gamma-aminobutyric acidergic) synaptic transmission, and FOS induction in the cocaine SA environment.

## METHODS AND MATERIALS

### Cell Culture

Primary cortical cultures were generated as previously described (34), with minor revisions (see Supplemental Methods for details), using P0 Sprague Dawley rats. Cells were infected with AAV2-3xFlag-HDAC5-WT and stimulated with forskolin. Analysis of HDAC5 localization was performed using immunocytochemistry Flag signal versus Hoechst stain for nuclear reference.

### Intravenous Drug SA

IVSA was performed as previously described (29) using Med Associates sound-attenuated operant chambers and SAI Infusion Technologies catheter supplies.

### Immunohistochemistry

Immunohistochemistry was performed as previously described (35). Images for quantitative experiments were collected with either the Stellaris Confocal Microscope or Thunder Imager (Leica). Placements were confirmed using either the Thunder Imager or Nikon Eclipse 80i fluorescent microscope.

### Electrophysiology

We prepared 300-μm coronal sections following rapid decapitation. Viral infection was visualized by co-infused enhanced green fluorescent protein (eGFP) virus. Evoked postsynaptic inhibitory/excitatory responses of neurons in layer V (L5) of the PrL were elicited by field stimulation with low-intensity pulses of stimulated current (25–100 μA, 100-μs duration). AMPA receptor–mediated excitatory postsynaptic currents (EPSCs) were recorded in voltage clamp mode at −70 mV, and inhibitory postsynaptic currents (IPSCs) mediated by GABA_A_ receptors were recorded in voltage clamp mode at 0 mV. To calculate the excitatory/inhibitory (E/I) (AMPA/GABA) ratio, the amplitude of AMPA response recorded at −70 mV was divided by the amplitude of the GABA response at 0 mV.

### RNA Sequencing

Tissue punches containing the PrL were extracted from virally infected tissue (indicated by eGFP signal) using a 2-mm biopsy punch. Tissue was processed for RNA extraction as previously described (29). Frozen samples were sent to BGI Americas Cooperation, and sequencing was performed using polyA mRNA directional RNA sequencing library preparation using a DNBSEQ platform.

## RESULTS

### HDAC5 Subcellular Localization Is Regulated by Synaptic Activity in Cortical Neurons

**Figure 1.**
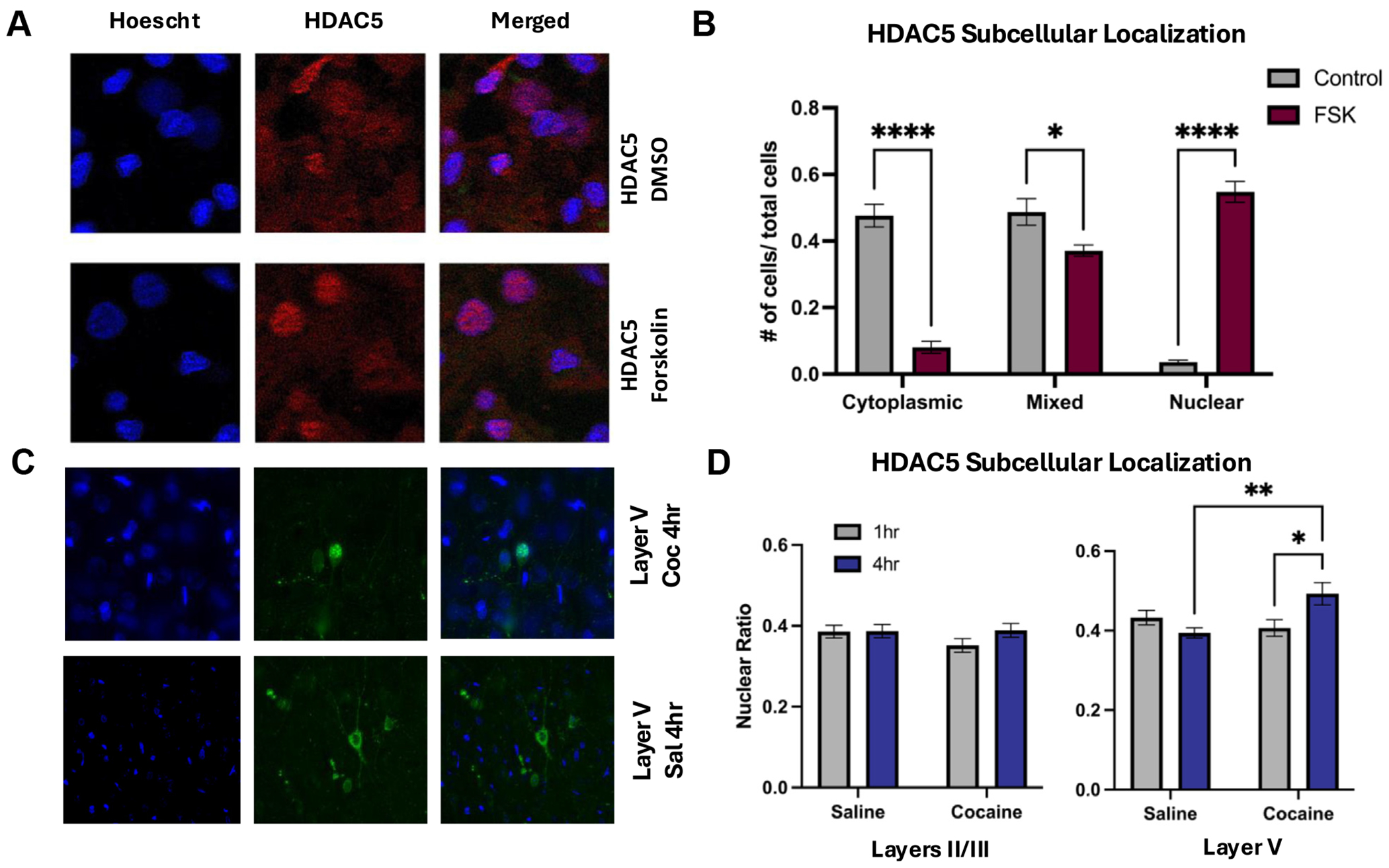
cAMP activation or cocaine self-administration promotes prelimbic HDAC5 nuclear accumulation. **(A, B)** In cultured cortical cells, HDAC5 is primarily located in the cytoplasm or mixed localization under control conditions. After a 3-hour incubation of 10 mm FSK to stimulate neuronal activity, HDAC5 is translocated to the nucleus (*n* = 3 slides per group, 53–76 cells per slide; 2-way ANOVA, Sidak’s multiple comparison test; *p < .05; ****p < .0001). **(C)** In vivo expression of HDAC5-wt-Flag in the prelimbic cortex of rats self-administering intravenous saline or cocaine. **(D)** HDAC5 translocated to the nucleus in layer V of cocaine self-administering rats 4 hours after cocaine (right) but not 1 hour after cocaine (left) (3 rats per group, 24–40 cells per condition; [left] 2-way ANOVA, not significant; [right] 2-way ANOVA, significant interaction, drug × time **p < .001, and Sidak’s multiple comparison test, *p < .05, cocaine 1 hour vs. 4 hours; **p < .01, 4 hours saline vs. cocaine). Data are shown as mean ± SEM. ANOVA, analysis of variance; cAMP, cyclic adenosine monophosphate; Coc, cocaine; DMSO, dimethyl sulfoxide; FSK, forskolin; HDAC5, histone deacetylase 5; Sal, saline.

In cultured primary cortical neurons, transfected wt-HDAC5 was observed primarily in the cytoplasm or in a mix of cytoplasm and the nucleus. Because drugs of abuse activate dopamine signaling in multiple addiction-related brain regions, we tested whether cortical HDAC5 is regulated by cAMP signaling. Similar to NAc neurons (27), increasing cAMP signaling with forskolin translocated HDAC5 to the nucleus (Figure 1A, B). We next infused a neurotropic AAV2-3xFlag-HDAC5-WT virus in the rat mPFC and assessed HDAC5 nucleocytoplasmic distribution following intravenous saline or cocaine SA. On the seventh day of IVSA, rats were sacrificed at 1 hour into the IVSA session (*t* = 1 hour) or 2 hours after the full 2-hour session (*t* = 4 hours). Consistent with previous findings (28) demonstrating delayed nuclear import of HDAC5 following cocaine exposure in the striatum, we also found a significant nuclear accumulation in L5 at 4, but not 1, hours after cocaine in the PrL (Figure 1C, D).

### HDAC5 in the PrL, but not IL, PFC Limits Context-Associated Cocaine Seeking

**Figure 2.**
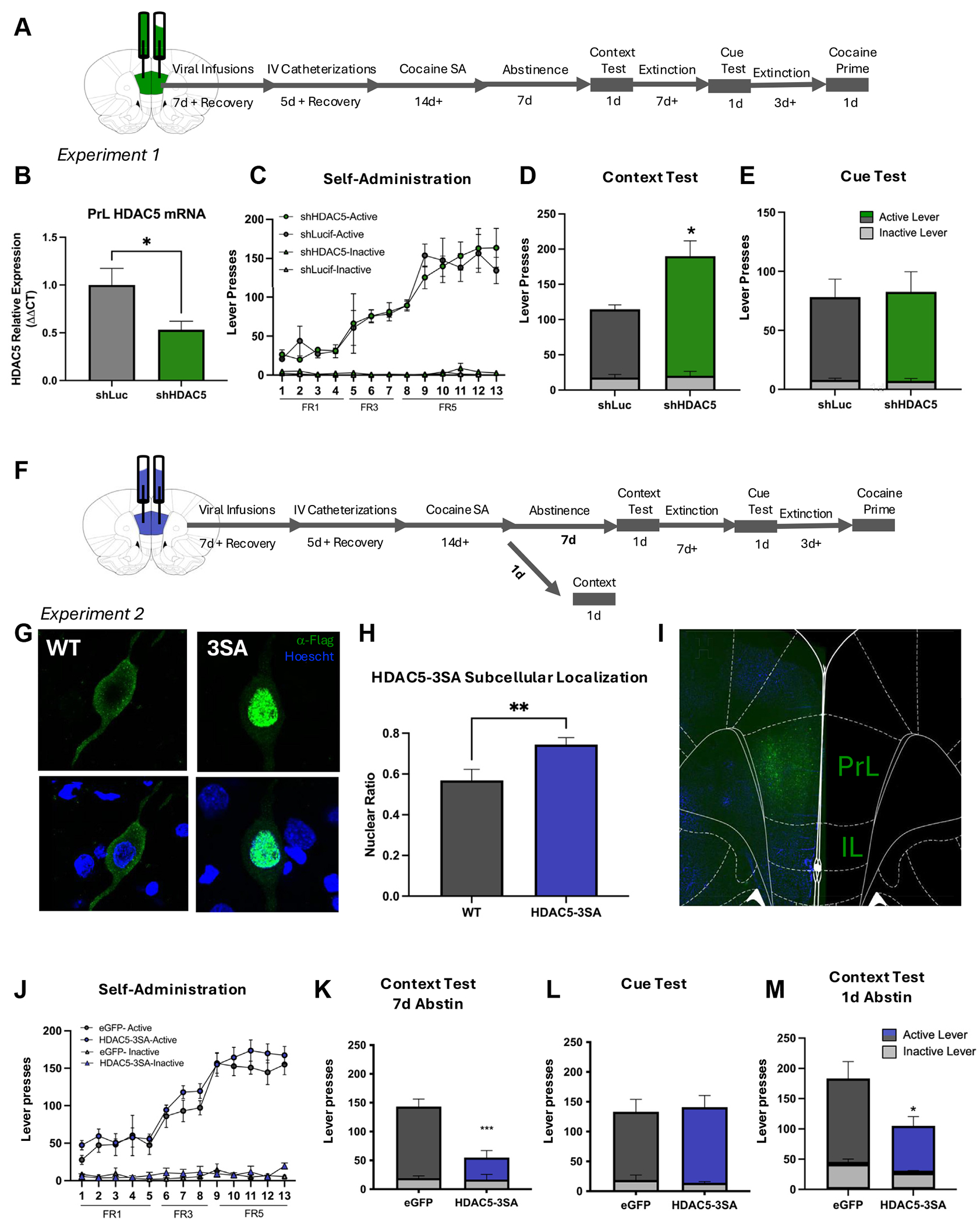
HDAC5 expression in the PrL bidirectionally regulates context-associated drug-seeking in a drug-specific manner. **(A)** Experiment 1 timeline. **(B)** Verification of AAV-shHDAC5–mediated reduction of endogenous HDAC5 mRNA expression in the PrL (*n* = 15 [7 shLuc, 8 HDAC5-3SA]; unpaired two-tailed *t* test; *p < .05). **(C)** HDAC5 reduction did not alter cocaine SA acquisition or stable cocaine-taking behavior (*n* = 15 [7 shLuc, 8 HDAC5-3SA]; 2-way repeated-measures ANOVA; not significant). **(D)** PrL shHDAC5 augmented context-associated cocaine-seeking behavior compared with AAV2-shLuc control condition (*n* = 15 [7 shLuc, 8 HDAC5-3SA]; unpaired two-tailed *t* test; *p < .05). **(E)** Following extinction training, there were no group differences in a cued reinstatement test (*n* = 15 [7 shLuc, 8 HDAC5-3SA]; unpaired two-tailed *t* test). **(F)** Experiment 2 timeline with target image of PrL HDAC5-3SA expression. **(G)** Representative staining of AAV2-CMV-3xFLAG-HDAC5-WT vs. AAV2-CMV-3xFLAG-HDAC5-3SA. Localization was quantified by immunohistochemistry Flag signal vs. Hoescht. **(H)** HDAC5-3SA shows increased nuclear localization in comparison with WT HDAC5 in the PrL. **p < .01. **(I)** Representative HDAC5-3SA histology in the PrL. **(J)** HDAC5-3SA expression did not alter acquisition behaviors (*n* = 29 [14 eGFP, 15 HDAC5-3SA]; 2-way repeated-measures ANOVA; not significant). **(K)** Following 7 days of abstinence, PrL HDAC5-3SA significantly suppressed context-associated cocaine seeking (*n* = 14 [7 eGFP, 7 HDAC5-3SA]; unpaired two-tailed *t* test; ***p < .001). **(L)** HDAC5-3SA expression had no effect on cue-reinstated seeking after extinction. **(M)** A subset of animals from experiment 2 underwent the context test after a 24-hour abstinence, and HDAC5-3SA significantly reduced cocaine seeking in the operant context (*n* = 15 [7 eGFP, 8 HDAC5-3SA]; unpaired two-tailed *t* test; *p < .05). Data are shown as mean ± SEM. Abstin, abstinence; ANOVA, analysis of variance; FR, fixed ratio; HDAC5, histone deacetylase 5; IL, infralimbic cortex; IV, intravenous; mRNA, messenger RNA; PrL, prelimbic cortex; SA, self-administration; WT, wild-type.

To investigate HDAC5’s influence in mPFC on cocaine IVSA behavior, we infused AAV2-shHDAC5 (29) into the PrL to reduce endogenous *Hdac5* mRNA (Figure 2B). Rats then performed IVSA on an increasing fixed-ratio schedule (Figure 2A). Compared with the control condition (AAV2-shLucif), viral-mediated HDAC5 knockdown in the PrL had no effects on cocaine IVSA acquisition, infusions, or stable drug taking (Figure 2C; Figure S1A). However, following 7 days of homecage abstinence, shHDAC5^PrL^ rats increased cocaine seeking under nonreinforced extinction conditions (Figure 2D), which we refer to as context-associated cocaine seeking. Following extinction training, in which the rats reduced pressing of drug-paired levers (Figure S1B), shHDAC5^PrL^ rats displayed cue-reinstated cocaine seeking and cocaine-primed drug seeking that was indistinguishable from control rats (Figure 2D; Figure S1C), suggesting that endogenous HDAC5 in the PrL limits context-associated cocaine seeking but not cued or drug-primed reinstatement of seeking.

We next asked whether increasing nuclear HDAC5 levels in the PrL could suppress context-associated cocaine seeking. We infused AAV2-3xFlag-HDAC5-3SA (28), a nuclear-localized mutant of HDAC5, or AAV2-eGFP bilaterally in the PrL of adult rats. Validation of HDAC5-3SA subcellular localization confirmed that HDAC5-3SA shows strong nuclear localization in vivo in the cortex (Figure 2G, H). Rats expressing HDAC5-3SA or eGFP self-administered cocaine, and we found no group differences in IVSA behavior (Figure 2J) or in total cocaine taking (Figure S1D). However, following 7 days of abstinence, HDAC5-3SA significantly reduced context-associated seeking (Figure 2K). We again detected no effects of PrL HDAC5-3SA overexpression on cued or drug-primed reinstatement of cocaine seeking (Figure 2L; Figure S1E, F). Further, the reduction in context-associated seeking produced by PrL HDAC5-3SA was not dependent on the 1-week of withdrawal because we also observed the effect after only 1 day of abstinence (Figure 2M). Together these findings provide bidirectional evidence suggesting a selective effect of PrL HDAC5 to limit context-related drug seeking.

**Figure 3.**
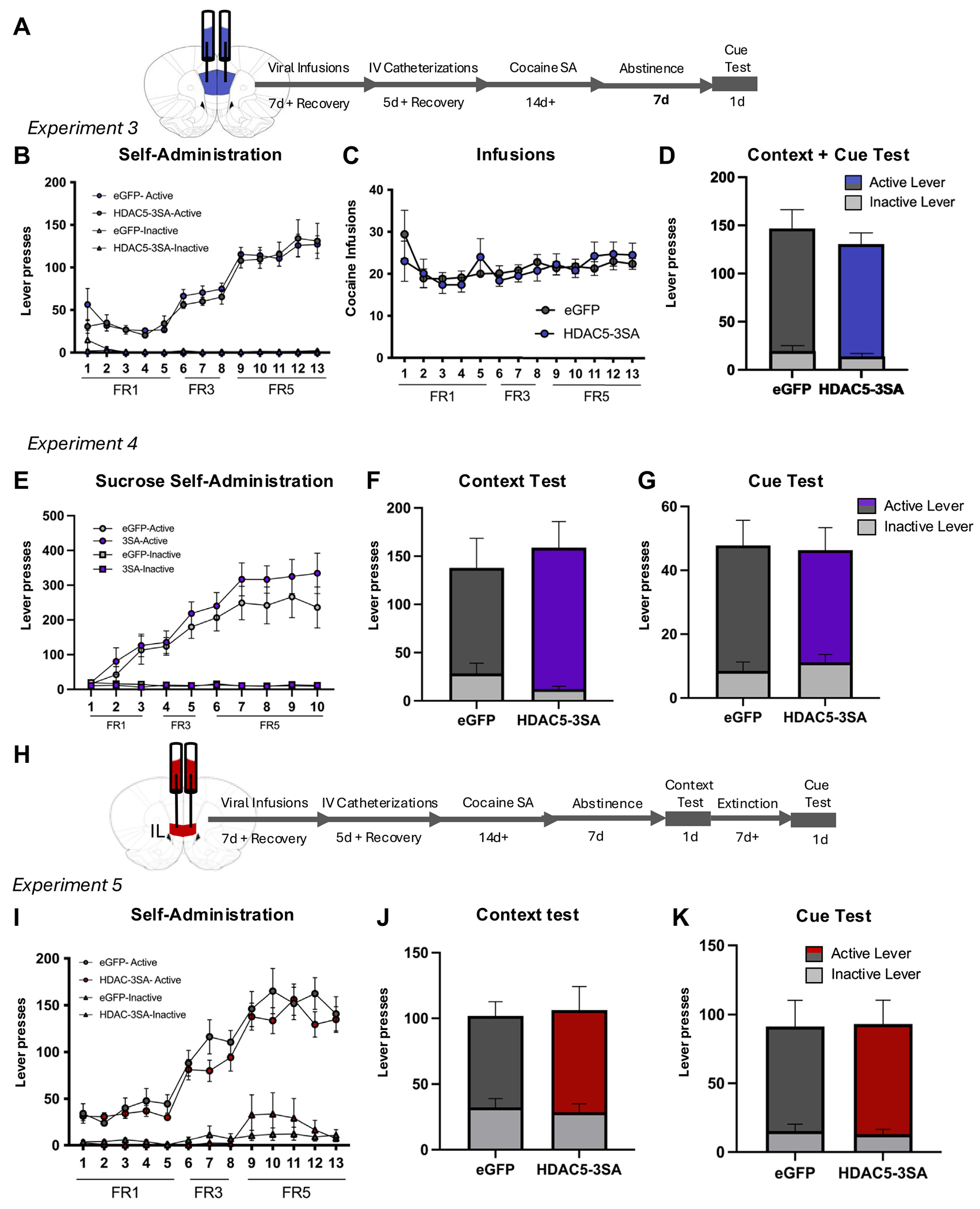
HDAC5-mediated reduction in context-associated seeking behavior is independent of extinction experience and drug and regionally selective. **(A)** Experimental timeline to test if extinction experience is necessary for HDAC5’s effect. There were no group differences in **(B)** SA or **(C)** total infusions (*n* = 17 [9 eGFP, 8 HDAC5-3SA]; 2-way repeated-measures ANOVA; not significant). **(D)** Following abstinence, we did not observe any differences in a context + cue relapse test (*n* = 17 [9 eGFP, 8 HDAC5-3SA]; unpaired two-tailed *t* test; not significant). **(E–G)** HDAC5-3SA in the PrL does not alter **(E)** sucrose SA behaviors, **(F)** context-associated seeking, or **(G)** cued reinstatement, indicating that HDAC5’s seeking suppression is drug dependent (*n* = 12 [6 eGFP, 6 HDAC5-3SA]). **(E)** Two-way repeated-measures ANOVA, not significant. **(F, G)** Unpaired two-tailed *t* test, not significant. **(H)** Experimental timeline. **(I–K)** HDAC5-3SA in the IL had no significant effect on acquisition, context test, or cue test seeking, indicating that the suppressive effect is PrL specific (*n* = 20 [11 eGFP, 9 HDAC5-3SA]). **(I)** Two-way repeated-measures ANOVA, not significant. **(J, K)** Unpaired two-tailed *t* test, not significant. Data are expressed as mean ± SEM. ANOVA, analysis of variance; eGFP, enhanced green fluorescent protein; FR, fixed ratio; HDAC5, histone deacetylase 5; IL, infralimbic cortex; IV, intravenous; SA, self-administration.

Extinction training involves discrete neural circuits in comparison with seeking following abstinence. In the previous experiments, HDAC5-3SA had no effect on cue-induced seeking, but cued seeking was only tested following extinction training. Therefore, we tested whether HDAC5-3SA could suppress cue-induced seeking with no extinction training (Figure 3A). As previously observed, HDAC5-3SA did not alter pressing behavior during SA or total infusions taken (Figure 3B, C). After abstinence, we returned rats to the chambers for a combined cue + context seeking test. We found no differences when cues were added to the context test (Figure 3D), indicating that HDAC5-3SA does not suppress cued seeking regardless of extinction experience.

To test whether PrL HDAC5-3SA limits general reward seeking, we next tested the effects of PrL HDAC5-3SA on sucrose SA and subsequent sucrose-seeking behavior. However, in contrast to cocaine IVSA, we detected no effects of PrL HDAC5-3SA on context-associated sucrose seeking or any other aspect of sucrose SA behavior (Figure 3E, G; Figure S2A, B), suggesting that HDAC5-3SA’s effects on context-associated seeking are drug specific.

The IL, a complementary mPFC region ventral to the PrL, plays an essential role in extinction of drug-seeking (17,36,37). To test whether HDAC5’s effect on drug seeking is specific to the PrL, we expressed AAV2-HDAC5-3SA or a control targeting the IL (Figure 3H). HDAC5-3SA expression in the IL had no effects on acquisition of cocaine IVSA, drug infusions, context-associated seeking, extinction, or cue-reinstated seeking (Figure 3I–K; Figure S3C, D). There was a trend for IL HDAC5-3SA to reduce cocaine-primed seeking, but it did not reach statistical significance (Figure S3E). Taken together, our findings show that HDAC5 functions selectively in the PrL to limit context-associated seeking.

**Figure 4.**
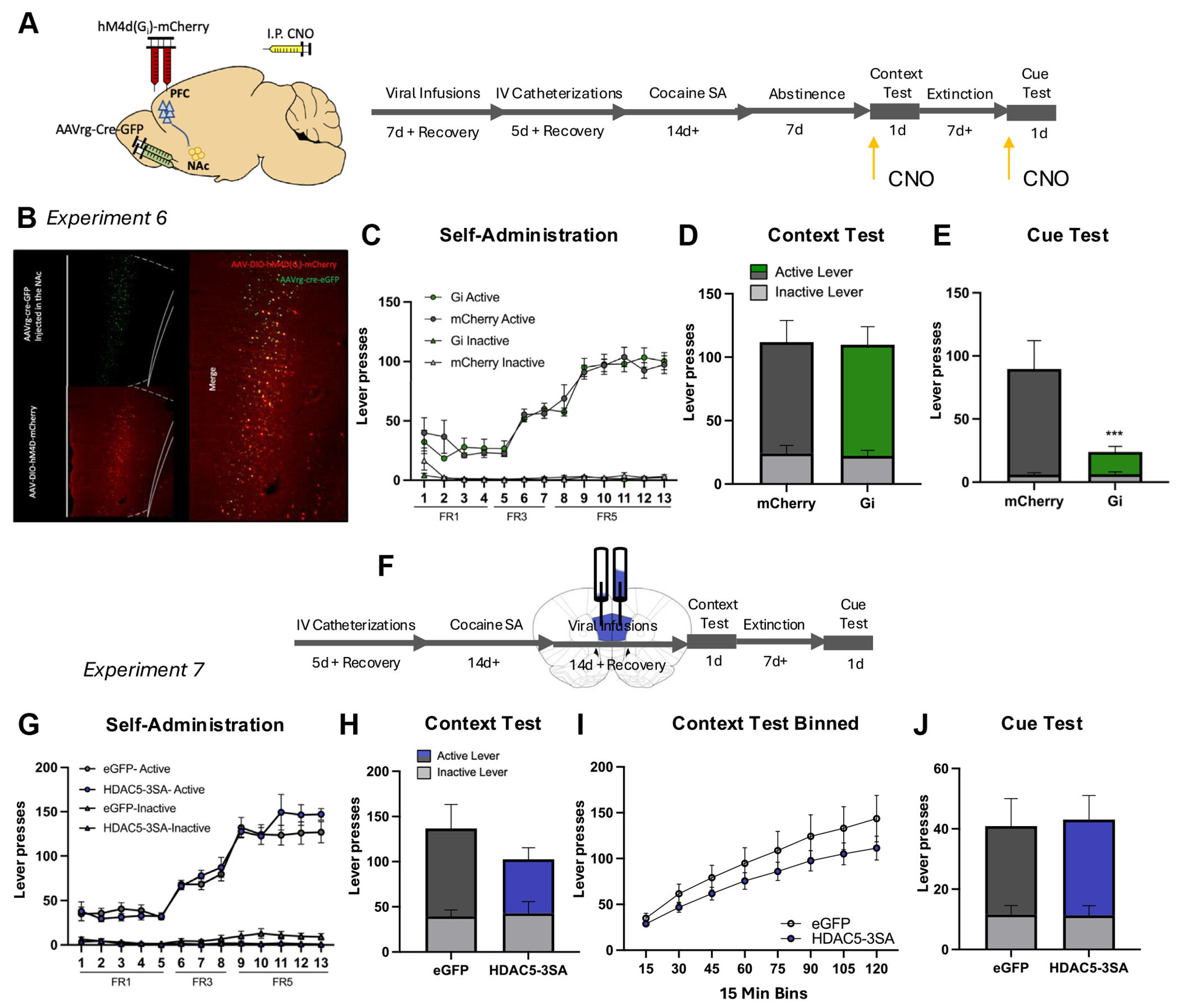
HDAC5-3SA acts selectively in the PrL, during acquisition of drug-taking behavior, to reduce context-associated seeking. **(A)** Experiment 5 timeline showing the pathway-specific chemogenetic viral strategy with hM4d(Gi) expression in NAc-projecting PrL neurons to assay the circuitry underlying context-associated seeking. **(B)** Representative expression of GFP-tagged AAVrg-Cre in NAc-projecting PrL neurons, mCherry-tagged hM4D + neurons, and overlay. **(C)** There were no group differences in IVSA behavior (*n* = 29 [14 mCherry, 15 hM4D-Gi]; 2-way repeated-measures ANOVA; not significant). **(D)** Following 7 days of abstinence, CNO-mediated inhibition of NAc-projecting neurons failed to alter context-associated seeking behavior (*n* = 29 [14 mCherry, 15 hM4D-Gi]; unpaired two-tailed *t* test; not significant) but **(E)** did significantly suppress cue-induced reinstatement following extinction to criteria (*n* = 29 [14 mCherry, 15 hM4D-Gi]; unpaired two-tailed *t* test; ***p < .001). **(F, G)** Experiment 6 timeline with cocaine self-administration acquisition prior to viral expression. In the 24 to 48 hours following the last day of stable cocaine SA, animals were infused with AAV-HDAC5-3SA and underwent 3 weeks of forced abstinence to allow for sufficient AAV expression. **(H)** HDAC5-3SA expressed following acquisition did not suppress context-associated cocaine seeking (*n* = 31 [16 eGFP, 15 HDAC5-3SA]; unpaired two-tailed *t* test; not significant), **(I)** with no effects observed across the full 2-hour session (in 15-minute bins) (2-way repeated-measures ANOVA; not significant) and **(J)** no effect on cue reinstatement (unpaired two-tailed *t* test; not significant), indicating that HDAC5-3SA works during drug acquisition to dampen context-drug associations. AAV, adeno-associated virus; ANOVA, analysis of variance; CNO, clozapine *N*-oxide; eGFP, enhanced green fluorescent protein; FR, fixed ratio; HDAC5, histone deacetylase 5; I.P., intraperitoneal; IV, intravenous; NAc, nucleus accumbens; SA, self-administration.

Because 1) bidirectional PrL HDAC5 manipulations had no effect on cue-reinstated seeking and 2) the PrL to NAc core (PrL→NAcore) circuit is required for cued reinstatement (15,38–41), we speculated that HDAC5 does not limit context-associated seeking through suppression of the PrL→NAcore circuit. However, a role of the PrL→NAcore circuit in context-associated cocaine seeking has not been tested. We used a pathway-specific, inhibitory chemogenetic approach (Figure 4A). Using a Cre-dependent, inhibitory DREADD (designer receptor exclusively activated by designer drugs) (DIO-hM4Di-mCherry, or DIO-mCherry control) in the PrL and retrograde, AAVrg-Cre-GFP in the NAc core, we selectively expressed Gi-DREADD in the PrL→NAcore neurons (Figure 4B). Following validation with ex vivo acute slice electrophysiology (Figure S3A), rats underwent cocaine IVSA and then received clozapine *N*-oxide (CNO) (5 mg/kg, intraperitoneal [i.p.]) to suppress PrL→NAcore circuit activity during the context test. Suppression of PrL→NAcore activity failed to alter context-associated seeking (Figure 4D), but in agreement with the prior literature (15,42), chemogenetic inhibition of the PrL→NAcore pathway in these same rats suppressed cue-reinstated seeking confirming the efficacy of our chemogenetic approach. Because our experimental design did not include a saline i.p. injection to control for the effects of CNO, these behavioral data must be interpreted in the context of potential CNO and its metabolites effects. These findings reveal that the PrL→NAcore is not required for context-associated cocaine seeking and that HDAC5 must limit this mode of seeking through a PrL→NAcore–independent circuit.

Because PrL HDAC5-3SA suppressed context-associated cocaine seeking at 1 day of withdrawal, we speculated that HDAC5 might be functioning during active cocaine SA to limit formation of drug-context associations. To test this idea, rats went through cocaine IVSA for 2 weeks and then we expressed HDAC5-3SA in the PrL and allowed 2 weeks for viral incubation (Figure 4F). Prior to virus infusion, there were no differences in IVSA behavior between assigned groups (Figure 4G; Figure S3D). Unlike the expression of PrL HDAC5-3SA throughout IVSA (Figure 2), we observed no effect of PrL HDAC5-3SA on context-associated cocaine seeking when expressed after completion of cocaine IVSA (Figure 4H). To ensure that we were not missing an effect on seeking, we analyzed 15-minute bins across the session but found no differences (Figure 4I). Like the PrL HDAC5-3SA study in Figure 2, the expression of PrL HDAC5-3SA after completion of cocaine SA had no effects on extinction or cue-induced reinstatement (Figure 4J; Figure S3E). Together, these results strongly suggest that PrL HDAC5 limits future context-associated seeking by antagonizing the formation and/or stability of the drug-context association produced during active drug use but without impacting the reinforcing effects of cocaine or operant discrimination learning and memory.

### HDAC5 Influences Expression of a Select Population of Cocaine SA–Regulated Genes

**Figure 5.**
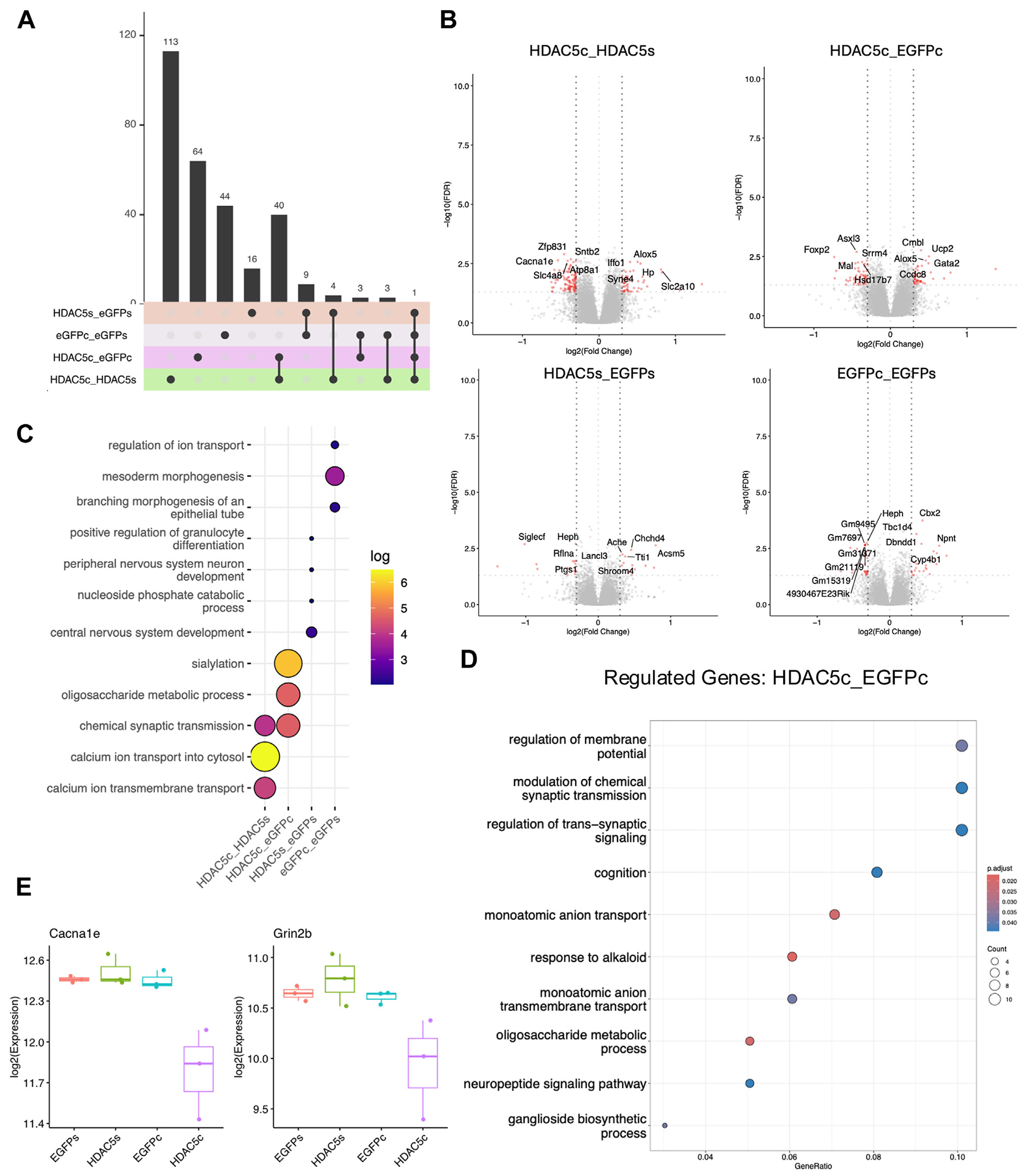
HDAC5 alters cocaine SA-induced gene expression in genes associated with excitatory and inhibitory neurons. **(A)** UpSet plot depicting overlapping differentially expressed genes between cocaine SA vs. saline SA groups, ± HDAC5-3SA. **(B)** Volcano plots showing the distribution of significant differentially expressed genes [FDR < 0.05; log_2_ (fold change) > |0.3|]. **(C)** Bubble chart depicting Gene Ontology analysis by group. **(D)** Bubble plot showing Gene Ontology analysis between HDAC5 and eGFP under cocaine conditions. **(E)** Box plot of expression levels of genes relevant to cocaine seeking and suppressed by HDAC5 following cocaine SA. eGFP, enhanced green fluorescent protein; FDR, false discovery rate; HDAC5, histone deacetylase 5; SA, self-administration.

As an epigenetic enzyme, we speculated that PrL HDAC5-3SA influences the formation of drug-context associations through regulation of gene expression. To explore this idea, we expressed HDAC5-3SA or GFP in the PrL and ran rats on cocaine or saline IVSA for 2 weeks followed by a 1-week abstinence period to match the behaviorally relevant time point just prior to the context-associated seeking test. Following bulk RNA-sequencing analysis of PrL tissues, we analyzed differentially expressed genes (DEGs) in 4 groups. eGFP-cocaine SA versus eGFP-saline SA produced only 44 significant DEGs (Figure 5A, B), and even fewer DEGs (i.e., 16) were produced by HDAC5-saline SA versus eGFP-saline SA rats. However, we detected the largest number of significant DEGs (i.e.,113) in the HDAC5-cocaine SA versus HDAC5-saline SA group, revealing an interesting interaction between cocaine SA and HDAC5 on gene expression. Similarly, HDAC5-cocaine SA produced 64 DEGs compared with eGFP-cocaine, again revealing an interesting interaction between HDAC5 and cocaine SA experience. Moreover, the majority of the HDAC5 DEGs were downregulated, suggesting that cocaine SA experience upregulates a population of genes and HDAC5-3SA represses the expression of a subset. Gene Ontology analysis revealed that HDAC5-cocaine had the strongest effect on modulation of chemical synaptic transmission relative to HDAC5-saline and eGFP-cocaine (Figure 5C), and under cocaine conditions, HDAC5-3SA reduced expression of glutamatergic signaling pathway genes (Figure 5D), indicating that the E/I balance may be affected. These downregulated DEGs (Figure 5E) include important neuronal plasticity genes, such as the NMDA receptor subunit *Grin2b* and a voltage-gated calcium channel *Cacna1e*. While the importance of these genes for drug-context associations remains unclear, the findings reveal an intriguing interaction between HDAC5 and cocaine SA on multiple plasticity genes in the PrL that might control the formation and/or stability of drug-context associations produced during active drug use.

### HDAC5 Enhances Synaptic Inhibition and Reduces SA Context–Induced Activation

**Figure 6.**
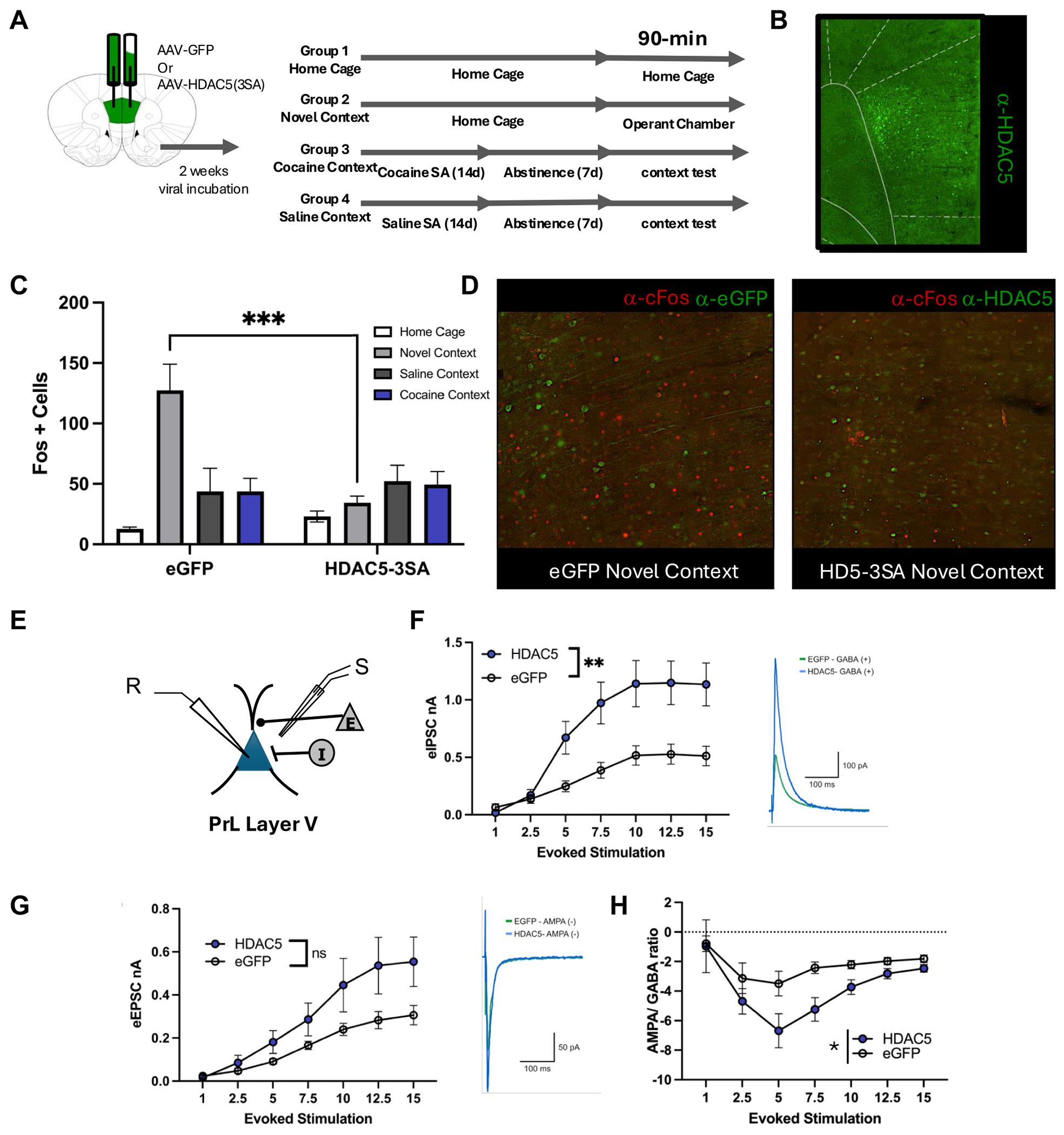
HDAC5 enhances GABAergic transmission on PrL pyramidal neurons and reduces FOS induction during novel environment exposure. **(A)** Experimental design for FOS neuronal activation assessment following exposure to a novel context, a context test (following either cocaine or saline SA), or a home cage control. **(B)** Representative expression of HDAC5-3SA. **(C)** HDAC5-3SA inhibited FOS expression only in animals exposed to the novel context (*n* = 4–6 per group; 2-way ANOVA, Sidak’s multiple comparison; ***p < .001). **(D)** Representative FOS expression following novel context exposure. **(E)** Experimental design of patch clamp electrophysiology in PrL neurons ± HDAC5-3SA. **(F)** Cortical HDAC5-3SA expression causes an increase in GABAergic tone in layer V pyramidal neurons, while not altering AMPA-related responses. Stepwise stimulation of pyramidal neurons revealed an increased IPSC amplitude (2-way repeated-measures ANOVA; group main effect, eGFP vs. HDAC5-3SA; **p < .01) but **(G)** no significant change in EPSC–evoked amplitude (*n* = 18 cells per group from 3 or 4 rats; 2-way repeated-measures ANOVA; group main effect, eGFP vs. HDAC5-3SA; ns [*p* = .092]). **(H)** This resulted in an overall decrease of AMPA/GABA ratio driven primarily by increased GABA evoked stimulation (2-way repeated-measures ANOVA; group main effect, eGFP vs. HDAC5-3SA; *p = .05). ANOVA, analysis of variance; E, excitatory; eEPSC, electrically evoked EPSC; eGFP, enhanced green fluorescent protein; eIPSC, electrically evoked inhibitory postsynaptic current; GABA, gamma-aminobutyric acid; HDAC5, histone deacetylase 5; I, inhibitory; ns, not significant; PrL, prelimbic cortex; R, recording; S, stimulation; SA, self-administration.

Because HDAC5 influences synaptic signaling genes, we next asked if HDAC5-3SA might impact the ability of the drug use context to activate the PrL. We measured the effects of HDAC5-3SA on the induction of the immediate early gene, *c-fos*, following introduction to the operant chamber as a proxy for neural activity in this region (43,44). Rats were infused in the PrL with AAV2-HDAC5-3SA or AAV2-eGFP control. We compared PrL FOS induction in SA-naïve rats taken from their home cage (group 1, baseline FOS levels), rats exposed to the SA operant chamber for the first time (group 2, novel context experience), rats trained to SA cocaine re-exposed to the chamber after 1 week of home cage abstinence (group 3, context-associated seeking test), and saline SA rats reexposed to the chamber after 1 week of abstinence (group 4, control group for cocaine SA) (Figure 6A, B). Interestingly, HDAC5-3SA had no effects on PrL FOS-positive density after cocaine or saline SA in the PrL, but it dramatically reduced the induction of the PrL FOS ensemble in the SA-naïve rats exposed to the operant chamber for the first time (group 2) (Figure 6C, D). Considering that HDAC5 selectively reduced context-associated seeking only when expressed during active cocaine SA, these findings suggest that PrL HDAC5 limits the strength of neural activity induced by the environment, which might be critical for synaptic plasticity supporting the formation of drug-context associations.

Because HDAC5-3SA reduced FOS expression when exposed to a novel context, but not at the point of the context test, we wanted to test the influence of HDAC5-3SA on the basal synaptic properties of PrL deep-layer pyramidal neurons (PNs) (considering we found selective nuclear localization in L5 in Figure 1B). We performed acute slice recordings of L5 PNs expressing HDAC5-3SA or eGFP only (Figure 6E). Using patch-clamp configuration, we observed that HDAC5-3SA produced a significant increase in electrically evoked IPSCs (eIPSCs) on L5 PNs (Figure 6F), but there was no significant effect of HDAC5-3SA on electrically evoked EPSCs (Figure 6G). Indeed, analysis of the AMPA/GABA ratio on L5 PNs revealed that HDAC5-3SA produced a significant main effect of HDAC5-3SA to reduce the E/I ratio (Figure 6H).

## DISCUSSION

A major obstacle in treating patients with SUDs is the persistent and prepotent nature of drug-cue and drug-context associations that can trigger a return to active drug use. However, there remains an incomplete understanding of the mechanisms by which drug experiences couple to the drug use environments. In the present study, we examined how the epigenetic enzyme HDAC5 acts within the mPFC to influence a highly relevant relapse-like behavior: drug seeking produced by re-exposure to the former drug-use environment. We found that reduction of endogenous HDAC5 in the PrL enhanced context-associated cocaine seeking without impacting acquisition of cocaine SA, extinction learning, or cued or drug-primed reinstatement of cocaine seeking. Viral-mediated overexpression of HDAC5-3SA, in the PrL but not in the IL, had the opposite effect by reducing context-associated cocaine seeking after 1 or 7 days of home cage abstinence, but again without altering other aspects of cocaine IVSA or reinstated cocaine seeking. PrL HDAC5-3SA had no effects on sucrose seeking, and it had no effects when manipulated after the establishment of cocaine IVSA, suggesting a selective role for HDAC5 in the formation and/or stabilization of drug—but not natural reward—context associations and drug seeking produced by exposure to the drug use environment. We also showed that, unlike cue-reinstated seeking, the PrL→NAcore pathway is not required for context-associated cocaine seeking, suggesting that HDAC5 influences a distinct PrL-dependent circuit(s) to influence this mode of relapse-like drug seeking. Analysis of HDAC5-regulated gene expression suggests that it impacted cocaine IVSA-regulated gene expression of several ontological gene groups linked to neural plasticity. Finally, we found that PrL HDAC5-3SA reduced the E/I ratio of synaptic transmission onto deep-layer PNs, largely by significantly increasing GABAergic inhibitory transmission, and it reduced the novel context-induced activation of the PrL, as measured by induction of the FOS-positive ensemble in this region. Taken together, our findings revealed a novel role for HDAC5 in the PrL, but not in the IL, to limit cocaine seeking, but not sucrose seeking, produced by re-exposure to a former drug use environment, and that the strength of drug-context associations is encoded, at least in part, by epigenetic mechanisms in the PrL.

We previously showed that HDAC5 functions in the NAc to limit drug-related behaviors, including cocaine CPP and cocaine- or heroin-seeking behaviors, including cue- and drug prime–reinstated seeking. Like the findings in the PrL, HDAC5 in the NAc does not alter any form of sucrose seeking (27,29). In striatal neurons, elevation of cAMP signaling by forskolin, activation of dopamine D_1_ receptors, or cocaine or heroin exposure induces a transient increase in nuclear HDAC5, which is produced by PP2A (protein phosphatase 2A)–dependent dephosphorylation of HDAC5’s conserved serines (27,29). The dephosphorylation-dependent nuclear accumulation in striatal MSNs is mimicked by the HDAC5-3SA mutant (i.e., S259A/S279A/S498A) (28), and our studies here reveal that HDAC5-3SA is nuclear-localized in mPFC neurons and that cocaine SA produces a modest increase in nuclear HDAC5. Because viral-mediated reduction of HDAC5 augments context-associated cocaine seeking and overexpression of HDAC5-3SA reduces it, our new findings show that HDAC5 functions in multiple SUD-related brain regions to limit relapse-like, drug-seeking behavior, thus positioning this epigenetic enzyme as a central regulator of neuroadaptations produced by repeated drug use that influence relapse vulnerability.

Transcriptional dysregulation following cocaine use is well documented in preclinical studies of the mPFC (23,45,46) and in the homologous region (dorsolateral PFC) in postmortem samples from patients with SUDs (47–50). Furthermore, human data indicate that cocaine use results in transient increased transcriptional activation followed by a refractory period of downregulation after the drug metabolism (49), and rodent studies report that withdrawal from cocaine results in upregulation of histone H3K9 acetylation in the mPFC and changes in associated gene expression (48). In the present study, we assessed the state of gene expression in animals after stable cocaine (or saline) SA and 1 week of abstinence to identify stable changes influenced by HDAC5 and/or cocaine SA. Moreover, this same time point represents the state of the PrL transcriptome just prior to re-exposure to the cocaine IVSA–paired environment, and because HDAC5 does not appear to function after cocaine SA to limit drug seeking, dynamic transcriptional changes produced during context-associated seeking seem less relevant to HDAC5’s function in this context. Interestingly, we only detected 44 DEGs in the comparison of eGFP-saline SA versus eGFP-cocaine SA, suggesting that cocaine SA plus 1 week of abstinence has a modest impact on PrL gene expression. However, in the HDAC5-3SA-cocaine SA rats, we observed larger effects on PrL gene expression compared with eGFP-cocaine SA (i.e., 64 DEGs) or compared with HDAC5-3SA-saline SA (i.e., 113 genes). Indeed, analysis revealed 2 intriguing DEGs where HDAC5-3SA reduces the expression only when animals had a history of cocaine SA, including *Grin2b*, which encodes the NMDA receptor subunit implicated in synapse plasticity, learning and memory, and cocaine use disorder, and *Cacna1e*, a voltage-gated R-type calcium channel subunit that gates calcium entry into neurons to influence their firing patterns (51,52). Future studies will be important to examine the functional relevance of these HDAC5-3SA DEGs, but they suggest a potential means by which HDAC5 might limit PrL plasticity supporting drug-context reactivity and context-associated cocaine seeking. It will be important to understand how HDAC5-3SA regulated increase in GABAergic synaptic transmission onto PrL L5 PNs and determine cell autonomous versus nonautonomous roles for HDAC5 in the PrL.

Exposure to environments in which drug use occurred is oftentimes unavoidable, and the circuitry of diffuse contextual cues underlying drug-seeking behavior is poorly understood. Many cocaine-seeking paradigms focus on reinstated cocaine-seeking after extinction (contextual or cued) (53–55). In these paradigms, the context-associated seeking test is considered extinction day 1 to adequately resolve cue-induced reinstatement from general IVSA context-associated seeking. Our study focused on this mode of relapse behavior—cocaine seeking triggered by re-exposure to a former drug-use environment under extinction conditions. There is surprisingly little known about the circuits involved in this form of drug seeking. The PrL→NAcore projection is well described as essential for discrete cue-reinstated drug seeking (15,40,42,56), but surprisingly, this projection is dispensable for cocaine seeking induced in the drug use environment under extinction conditions. Previous studies indicate that regions underlying extinguished contextual seeking and contextual seeking after abstinence are distinct. For example, inactivation of the dorsomedial PFC prevents context-induced cocaine seeking after extinction in a paradigm in which extinction training occurs in a distinct environment but not following abstinence, as tested here (56,57). Importantly, because we administered CNO intraperitoneally, neurons with collateral projections were also inhibited. Therefore, the role of these projections, including the basolateral amygdala, midline thalamic nuclei, and contralateral cortical projections, cannot be ruled out (58,59). However, our finding that chemogenetic inhibition of the PrL→NAcore pathway did not reduce context-associated seeking leaves the essential circuitry underlying context cocaine seeking an important target for future investigations. An interesting target would be hippocampal to PrL inputs because hippocampal lesions reduce measures of context-associated memory like fear conditioning (60,61). Activation of ventral hippocampus (vHipp)→PrL neurons reduces context fear retrieval (62). Furthermore, vHipp activation altered the PrL transcriptome, presenting the possibility that HDAC5-mediated changes in gene transcription may interfere with context-encoding in a similar manner. Furthermore, Twining *et al.* (63) showed that suppression of vHipp→PrL neurons reduced context-associated, but not cue-associated, fear memory, presenting an interesting parallel to the present finding that PrL HDAC5 suppressed context-associated, but not cue-associated, seeking. Future studies on the role of these projections may provide insights into context seeking.

Our findings here strongly suggest that PrL HDAC5 functions during cocaine SA training to limit the strength of the drug-taking environment to stimulate cocaine seeking because manipulation of HDAC5 after acquisition had no effects. These results are strengthened by our finding that PrL activation, as measured by FOS+ cells, was not different between HDAC5-3SA–expressing rats and control rats following context-associated seeking but was different following first exposure to the SA chambers. This might suggest that cocaine experience in the drug-taking chambers induces plasticity during early acquisition, and that HDAC5 limits that process suppressing the initial activation, and possibly plasticity, of the PrL ensembles [similar to Anderson *et al.* (29)]. Future studies are needed to understand how drug-context memories are formed and regulated by PrL circuits and the role of HDAC5 to limit drug-context associations.

Taken together, our findings show that HDAC5 acts within the PrL to enhance inhibitory tone and modulate cocaine-induced gene expression, resulting in dampened context-drug associations and reduction of future context-associated cocaine seeking. These data provide important insight into an understudied modality of drug seeking: the unextinguished drug use context. Future studies will focus on understanding the circuitry involved in unextinguished context-associated seeking and determining the important regions upstream and downstream of the PrL in this form of drug seeking.

## Supplementary Material

1

Supplementary material cited in this article is available online at https://doi.org/10.1016/j.biopsych.2025.06.027.

## Figures and Tables

**Table:** 

Resource Type	Specific Reagent or Resource	Source or Reference	Identifiers	Additional Information
Add additional rows as needed for each resource type	Include species and sex when applicable.	Include name of manufacturer, company, repository, individual, or research lab. Include PMID or DOI for references; use “this paper” if new.	Include catalog numbers, stock numbers, database IDs or accession numbers, and/or RRIDs. RRIDs are highly encouraged; search for RRIDs at https://scicrunch.org/resources.	Include any additional information or notes if necessary.
Other: Single use catheter	Catheter access button	SAI infusion technology	Sku # CAB22-R1	
Other: IVSA drug tether	Catheter tether	SAI infusion technology	CAB22-T12-P1	
Antibody	Alexa Fluor 488 AffiniPure Donkey anti-Chicken	Jackson ImmunoResearch Labs	Cat#: 703-545-155; RRID:AB_2340375	
Antibody	Alexa Fluor 594 AffiniPure Donkey anti-Mouse	Jackson ImmunoResearch Labs	Cat#: 715-585-150; RRID:AB_2340854	
Antibody	Alexa Fluor 488 AffiniPure Donkey anti-Mouse	Jackson ImmunoResearch Labs	Cat#: 715-585-150; RRID:AB_2340854	
Antibody	chicken anti-GFP	Aves Labs	Cat# GFP-1020; RRID:AB_10000240	
Antibody	Donkey anti-Mouse IgG, Alexafluor 488 conjugated	Thermo Fisher Scientific	Cat#: A-21202; RRID:AB_141607	
Antibody	mouse anti-FLAG M2	Sigma-Aldrich	Cat# F1804; RRID:AB_262044	
Chemical Compound or Drug	Aquamount	Epredia	Cat# 14-390-5	
Chemical Compound or Drug	Brevital	Par Pharmaceutical	Cat# 42023010501	
Chemical Compound or Drug	Cefazolin	MWI Animal Health	Cat# MWI 008902	
Chemical Compound or Drug	Cocaine hydrochloride	RTI International	N/A	NIDA Drug Inventory Supply and Control System
Chemical Compound or Drug	Heparin Lock Flush	Medline	Cat# 63323-545-05	
Chemical Compound or Drug	Hoescht 33342	Invitrogen	Cat# H3570	
Chemical Compound or Drug	Ketofen	MWI Animal Health	Cat# MWI 002800	
Chemical Compound or Drug	DMEM	Thermo Fisher Scientific	Cat# 11-960-044	
Chemical Compound or Drug	Neurobasal	Thermo Fisher Scientific	Cat# 21-103-049	
Chemical Compound or Drug	Papain	Worthington	Cat# LS003126	
Chemical Compound or Drug	Prolong Gold	Invitrogen	Cat# P36930	
				
Organism/Strain	Sprague-Dawley Rats	Charles River	RRID: RGD_734476	
Recombinant DNA, Viral Strain	AAV-CMV-3XFLAG-HDAC5-3SA	Plasmid:This Paper, Virus: University of South Carolina Viral Vector Core	N/A	
Recombinant DNA, Viral Strain	AAV-CMV-3XFLAG-HDAC5-3SA-CSCS	Plasmid:This Paper, Virus: University of South Carolina Viral Vector Core	N/A	
Recombinant DNA, Viral Strain	AAV-CMV-3XFLAG-HDAC5-CSCS	Plasmid:This Paper, Virus: University of South Carolina Viral Vector Core	N/A	
Recombinant DNA, Viral Strain	AAV-CMV-3XFLAG-HDAC5-WT	Plasmid:This Paper, Virus: University of South Carolina Viral Vector Core	N/A	
Recombinant DNA, Viral Strain	AAV-GFP	Plasmid PMID: 28957664, Virus: University of South Carolina Viral Vector Core	N/A	
Recombinant DNA, Viral Strain	AAV-shLuciferase	This Paper	N/A	
				
