# Supplementary material for: Histone Deacetylase 5 in Prelimbic Prefrontal Cortex Limits Context-Associated Cocaine Seeking
Source: Biol Psychiatry. Author manuscript; Available in PMC 2026 Apr 14. (PMC13072437; doi:10.1016/j.biopsych.2025.06.027)
Supplement: Supplementary file 1 [file NIHMS2158850-supplement-1.pdf]

SUPPLEMENTARY INFORMATION

Histone Deacetylase 5 in Prelimbic Prefrontal Cortex Limits Context-Associated Cocaine Seeking

Barry *et al.*

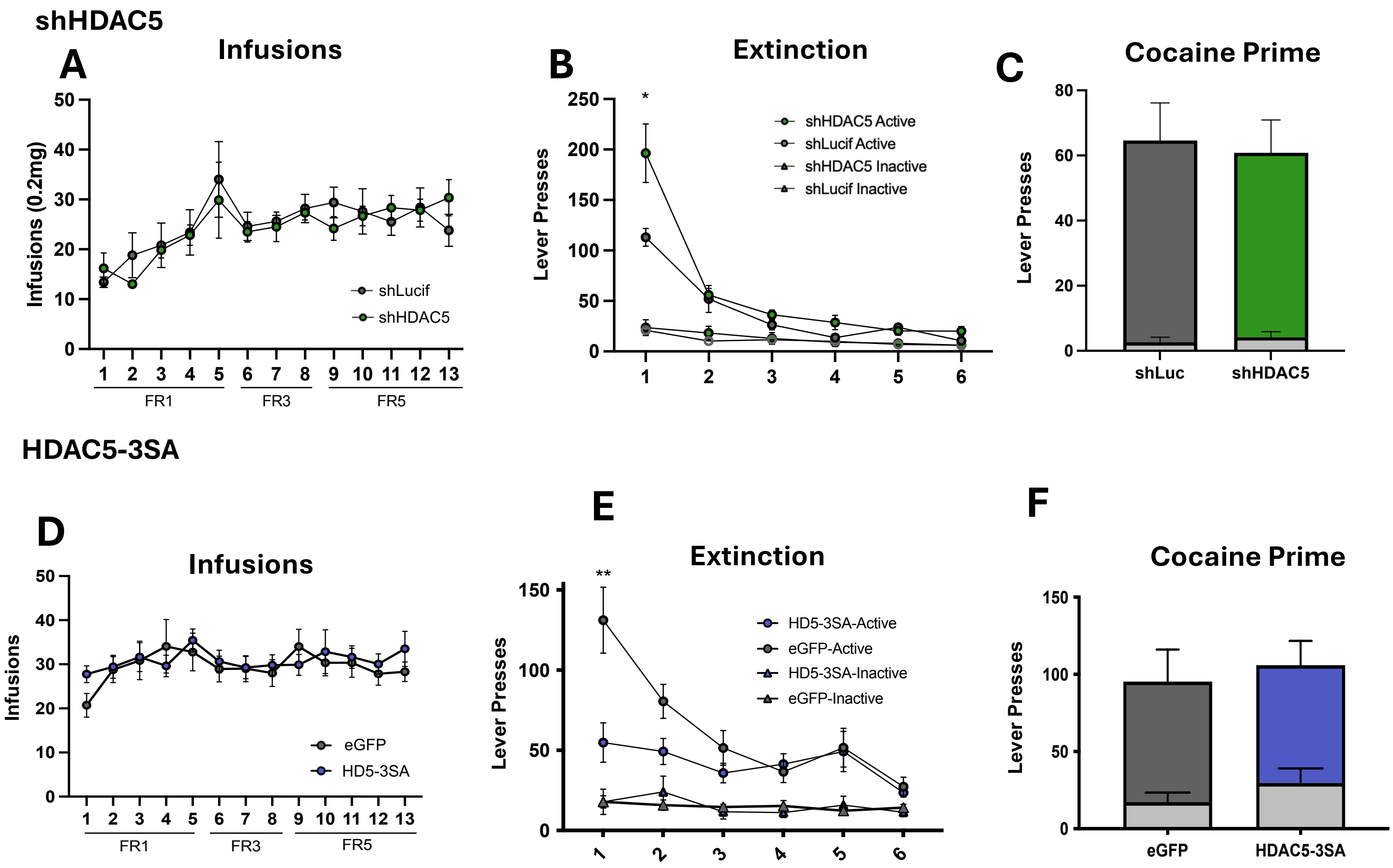

Supplemental Figure 1

Supplemental Figure S1. shHDAC5 expression did not alter total cocaine intake during acquisition (**A**) or alter extinction behavior (**B**) or cocaine-primed reinstatement (**C**). HDAC5-3SA expression in the PrL did not alter infusions, extent of extinction, or cocaine-primed reinstatement (**D-F**).

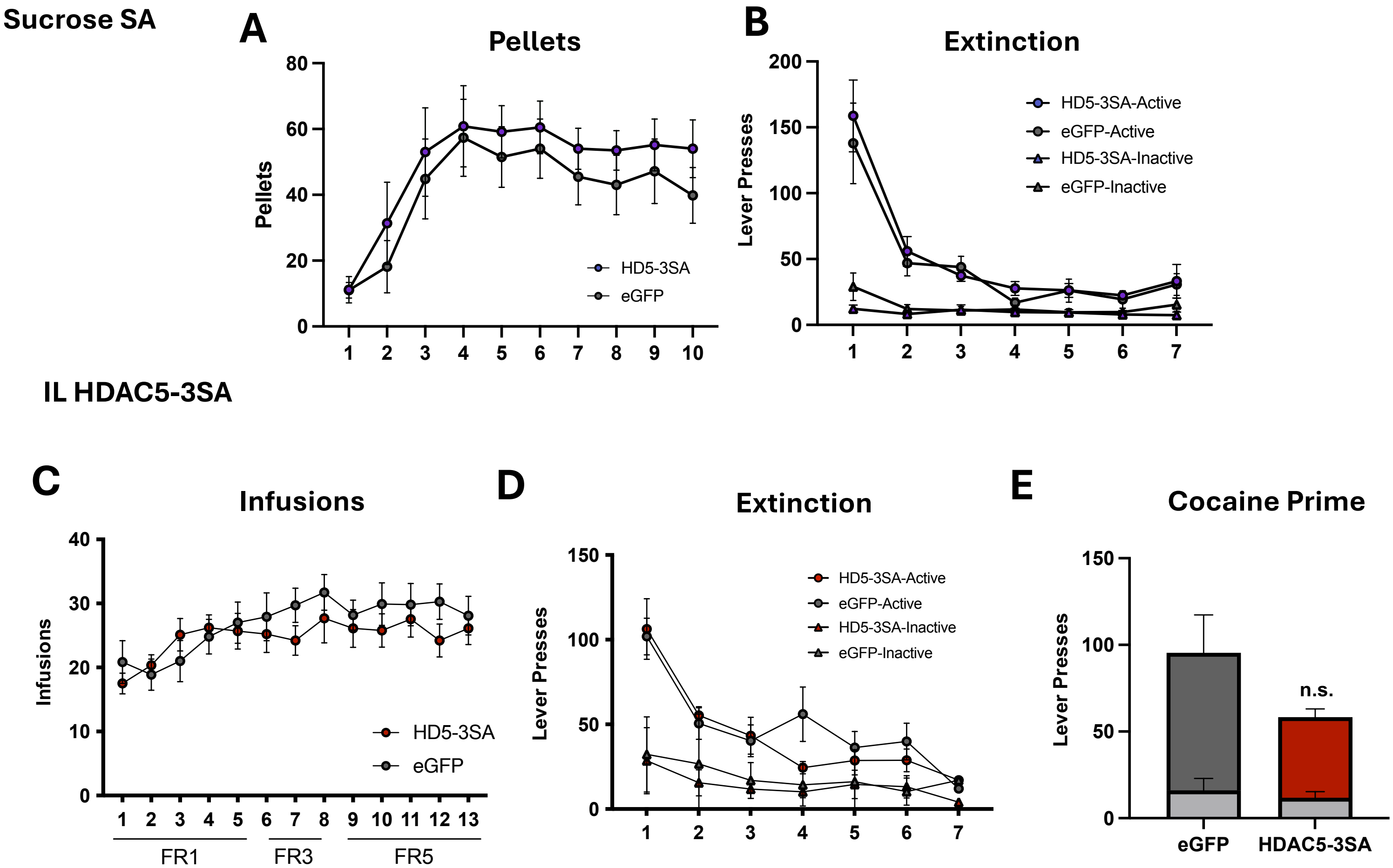

Supplemental Figure 2

Supplemental Figure S2. HDAC5-3SA expression did not alter total sucrose pellets administered in sucrose SA (**A**) or lever-pressing during extinction training (**B**). HDAC5-3SA expression in the IL did not alter total cocaine infusions or extinction behavior (**C-D**) . There was a statistical trend for a decrease in cocaine-primed seeking behavior, but these data failed to reach significance (unpaired two tailed t test, n.s n= 18[10 eGFP, 8 HD5-3SA**)** (**E**).

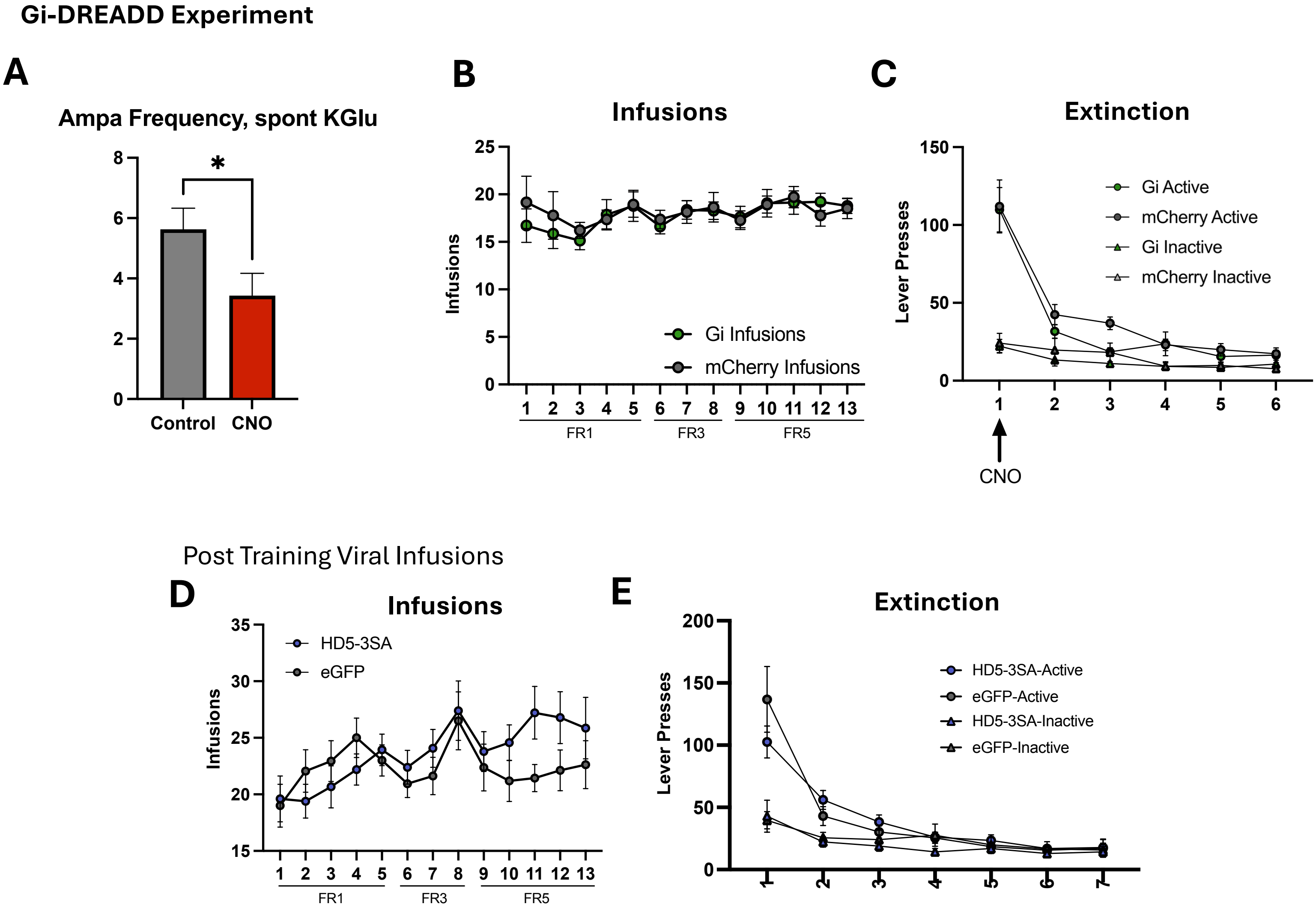

Supplemental Figure 3

Supplemental Figure S3. Acute slice electrophysiological validation of hM4d(Gi) DREADD efficacy in PrL pyramidal neurons showed a reduction in spontaneous AMPA frequency (**A**). Viral mediated DREADD and control expression did not alter overall cocaine intake or extinction behavior (**B-C**). Prior to infusion with HDAC5-3SA, there were no differences in cocaine infusions (**D**) or subsequent extinction behavior (**E**).

Supplemental Material & Methods

Cell Culture and Transfection: (**Fig1A**): Primary cortical neurons were extracted from Sprague Dawley rats (P0) Rats (Charles River) in concentrated dissecting media diluted (1:10 in HBS). Cells were then incubated in enzymatic solution (papain/ DNAase/ DMEM, 400µl, 100µl, q.s. 50mls DMEM) for 10 mins and washed 2x times with autoclaved distilled H_20 and then incubated 3x for 5mins in inhibitor solution (250µl DNA-ase/ 0.68 grams trypsin inhibitor/ q.s. 50ml DMEM). Cells were then washed 3x timles with warmed DMEM, gently mixed, counted, and plated (1 million cells per plate) onto coated 12 well plates in DMEM (incubated with poly-d-lysine and DMEM [1:100 PDL to DMEM] for 3 hours) on wells outfitted with coverslips (incubated in 10N HCL for 2 weeks then autoclaved to allow for neuronal adhesion). All solutions sterile filtered (0.2µm). Plated cortical neurons were infected with AAV2-3xFLG-HDAC5-WT at DIV-8 ,with media replaced 4 hours following infection. Cultures were were stimulated with 10um of Forskolin or DMSO and incubated for 3 hours at DIV-10. See Key Resource Table for product info.

Subcellular Localization Analysis:

In vitro Analysis: (Fig 1A) ~ Stimulated cell cultures were then washed with 0.5mls of ice-cold 1xPBS and fixed with 5mls of 4% formaldehyde, 2% sucrose in PB, incubated for 20 mins, and washed 3 times with PBS. Cells were permeabilized and blocked with 0.4% Triton X-100, 1% normal donkey serum in TBS-Tween 0.05% for 1 hour at room temperature and washed twice with PBS. Mouse-Anti-Flag in 3% BSA TBST (0.05%) was added and incubated overnight at 4C, and washed 3x with PBS. Then wells were incubated with anti-mouse-594 in 3% BSA TBST (0.05%) for 3 hours at 4C. Hoechst stain (1:10,000) was added in PBS and incubated for 1 min at RT following by 3x PBS washes. Coverslips were mounted on microscope slides and secured with Aquamount and imaged on Stellaris Confocal Microscope (Leica). Localization was determined by comparing Flag signal with Hoechst counter stain at 40x power. Cells were categorized as nuclear, cytoplasmic, or mixed. Localization is expressed as the percentage of each subcellular localization of the total cells measured.

In vivo Analysis (Fig 1B)*:* Rats expressing 3x-Flag-HDAC5-3SA or WT in the PrL were transcardially perfused with cold 4% paraformaldehyde and 1xPBS flush. 60um sections were taken on a Leica Vibrating Microtome. See below for IHC and antibody details. Images were acquired with a Zeiss LSM 880 confocal microscope equipped with a 63x oil immersion objective (1.4 NA), 0.2um step size, 1.5x digital zoom. Images were then deconvolved with Zen Black (v14, Carl Zeiss) with automatic filtration settings. Images were then imported into Imaris (v9, Bitplane). A surface was built around the nucleus using the DAPI signal. We then expanded that surface by 2.5 um to make a new surface (cytoplasm) using the “Dilate-Surfaces” MatLab extension. We found this expansion amount most accurately encompassed the cytoplasmic area of the cell without including neighboring cells or dendrites. We then created a surface of the nuclear flag signal (total

HDAC5 signal in the nucleus) and a surface of the cytoplasmic flag signal (total HDAC5 signal in the cytoplasm) by masking out flag signal outside the selected surface. The intensity sum of each region was divided by the volume of that region and expressed as the proportion of cytoplasmic to nuclear signal . $\left(\frac{Cyto-Flag\ Intensity}{Cyto\ Surface\ Volume}\right) / \left(\frac{Nuclear-Flag\ Intensity}{Nuclear\ Surface\ Volume}\right)$.

Animals: Male Sprague Dawley rats, singly housed upon arrival, aged 6-8 weeks and weighing 275-325g at delivery were kept in a climate-controlled vivarium on a reverse light dark cycle to allow for testing during their active period. Prior to experimental procedures, animals were habituated for a minimum of 3 days and allowed *ad libitum* access to standard chow and water. Animals were 7-9 weeks old at the time of first surgery. During the contingent self-administration portion of the experiments, animals were restricted to 20g of chow per day, or approximately 85% of their free feeding weight. All experimental procedures were approved by the Institutional Animal Care and Use Committee at Medical University of South Carolina, and in accordance with NIH and National Research Council guidelines.

Viruses:
Endogenous HDAC5 expression in the PrL was knocked down with AAV2-shHDAC5-GFP: shHDAC5 Sequence: GAAGGTTCTACAGAGAGCGAG under a U6 promoter, GFP under cytomegalovirus (CMV) promoter produced by the University of South Carolina Vector Core (titer 1.2 X 10^{12}, previously published(1, 2)), compared to control AAV2-shLuc-GFP*:* Sequence: CTTACGCTGAGTACTTCGA under a U6 promoter, GFP under cytomegalovirus (CMV) promoter produced by the University of South Carolina Vector Core (titer 1.2 X 10^{12}). We used the same *AAV2-HDAC5-3SA:* Promoter: CMV, as previously described (3), with an added N-terminal Flag- tag for optimized IHC (titer 6.77x10^{14}), and compared to AAV2-GFP(titer 6.07X10^{14}), both produced by the University of South Carolina Vector Core. Chemogenetics were conducted using either AAV5-hSyn-DIO-hm4d-mCherry (AddGene #44362-AAV5, titer 1.4x10^{13}), AAV5-hsyn-DIO-mCherry (AddGene, #50459-AAV5, titer 1.6x10^{13}) in the PrL and AAVrg-EGFP-cre (AddGene #105540-AAVrg, titer 1.17x10^{13}).

Drugs:
<u>CNO *N*-Oxide</u> (Tocris, #4936, reconstituted with 0.9% (w/v) normal saline to 5.0 mg/ml, light protected until i.p. injections).
<u>Cocaine-HCL</u> (NIDA Drug Supply Program) was dissolved in sterile 0.9% (w/v) saline to a final concentration of 4mg/ml to be administered at a dose of 0.5mg/kg/infusion in the rat IVSA paradigm.

Surgical Procedures:
Intracranial Infusions: Viral infusions were delivered stereotaxically as previously described[61]. Rats were anesthetized using 5% Fluriso™, isoflurane (Vet One), and maintained under deep anesthesia at 2-3%. Supportive heat and fluids were provided. Ketoprofen (5mg/kg, s.q.) was

administered pre-operatively and cefazolin (10mg/kg s.q.) were administered post-operatively (Patterson Veterinary Supply, Devens MA). Viruses were infused bilaterally into the PrL (+2.8 A/P, +/- 0.62mm M/L, -3.8mm D/V), IL (+2.8 A/P, +/- 0.64mm M/L, -5.5mm D/V or NAc Core (10º angle; from Bregma: +1.6 A/P, +/- 2.8 M/L, -7.1 D/V) with Nanoject Auto-nanoliter Injectors (Drummond Scientific Company, Broomall, PA), and pipettes were left in place for 5 minutes to allow diffusion of virus.

Catheter Implantation: Catheter implantations were performed as described previously(4). In brief, on end of a silastic catheter (Thermo Fisher Scientific or SAI Infusions Technologies, Lake Villa, IL) is inserted into the right jugular vein, secured, and threaded subcutaneously to a back mounted infusion cannula (Plastics One, Roanoke VA) or Catheter Access Buttons™ with Cannulock™ (SAI Infusions Technologies, Lake Villa, IL).

Cocaine IVSA: As previously described(4), rats were trained to self-administer cocaine intravenously in operant chambers (Med-Associates) on an escalating FR schedule (FR1, 3, 5) for 2-hr sessions for approximately 12-14 days. Rats were trained to press an active lever to receive an infusion of cocaine HCL (0.5mg/kg/infusion) paired with a light-tone cue complex. Following a reinforced active lever press, a 15 second time out occurred where presses were recorded but not rewarded with drug infusion. Inactive lever presses were recorded but not reinforced. Self-administration criteria was set at a minimum of 10 cocaine infusions for five days (FR1), 3 days (FR3), and 5 days (FR5). Following completion of FR training, rats were subjected to a period of abstinence in the vivarium (24hrs or 7 days). Following abstinence, rats were returned to the chambers under extinction conditions. Lever presses were recorded but not reinforced with drug or cue complex presentation. When rats reached the extinction criteria of a minimum of 7 days of extinction training and less than 25 active lever presses for 2 consecutive days, they underwent a cue-induced reinstatement test, whereupon pressing of the active lever resulted in the presentation of the cue+tone complex but not a drug infusion. If rats did not extinguish after 14 days, they were not included in further studies (n=2, experiment 5) In some experiments, rats were then extinguished to criteria and given a cocaine prime (10 mg/kg, i.p.) prior to being placed in the SA chamber and responses were recorded under extinction conditions. Rats were removed from the study if their catheters failed twice or if viral expression was outside of the intended region or unilateral.

General Immunohistochemistry: Following cessation of testing, rats were anesthetized and transcardially perfused with 150 mls of ice-cold phosphate buffered saline (1X PBS) followed by 4% (w/v) PFA (in PBS) at a rate of 20 ml/min. Brains were then post-fixed for 24-hrs in 4% (w/v) PFA (in PBS) and transferred to 30% (w/v) sucrose (in PBS) for cryoprotection, and then sectioned (40um) on a freezing microtome. Sections were then blocked with 3% NDS or NGS (Jackson Imm, 0.3% (v/v) Triton X-100, 0.2% (v/v) Tween-20, 3% (w/v) BSA for 3 hours at RT, and then incubated O/N at 4°C with blocking solution and primary anti-c-Fos (226 008, Synaptic Systems, rabbit, 1:500), anti-mCherry (NBP2-25157 Novus Biologicals, rabbit, 1:1000), anti-Flag (50-165-

7385 Sigma Aldrich, mouse, 1:1000), anti-GFP (Aves, GFP-1020, GRP-1026, chicken, 1:10,000, serial diluted), anti-HDAC5 (2082BV, Cell Signaling, Rabbit 1:200, Figure 1 only), anti-HDAC5-35 (Mouse, ab50001 abcam, Mouse (1:500-1:1000) and gentle agitation. Sectioned were then washed 3x 5mins in 1x PBS and incubated for 1 hour under light protected with Alex Fluor® 488 donkey anti-chicken (703-545-155, 1:500, Jackson ImmunoResearch), Alex Fluor® 488 donkey anti-mouse (715-586-150, 1:500, Jackson ImmunoResearch), Alex Fluor® 594 donkey anti rabbit (711-587-003, 1:500, Jackson ImmunoResearch). Sections were washed 3x 5mins in 1xPBS, then incubated with Hoechst 33342 (H3570, 1:10,000 (serially diluted), Invitrogen) for 1 min, washed twice more, then mounted on SuperFrost+ with ProGold Antifade or FluoroGold mount.

Electrophysiological Procedures: Acute coronal slices (300-μm thickness) containing the PrL cortex were obtained using a Leica VT1200s slicer in semi-frozen artificial cerebrospinal fluid (ACSF) containing (in mM): 127 NaCl, 2.5 KCl, 1.2 NaH_2PO_4, 24 $NaHCO_3$, 11 D-glucose, 1.2 $MgCl_2$, and 2.40 $CaCl_2$, 0.4 Na-ascorbate (pH 7.4, 315-320 mOsm). Kynurenic acid (5 mM) was added to the ACSF to avoid overactivity of glutamatergic receptors during recording. Slices were then transferred to normal ACSF (without kynurenic acid) to recover at 37°C for 30 minutes, and then transferred to room temperature ACSF for an additional 30-minute recovery, followed by recordings. All solutions were continually equilibrated with 95% O_2 and 5% CO_2. Pyramidal neurons in Layer V (LV) of PrL were tested and visualized with infrared differential interference contrast optics (DIC/infrared optics) and identified by their location and shape of the neurons and their apical dendrites. Unless stated otherwise, all electrophysiological experiments were performed in whole cell voltage clamp mode at -70 mV using borosilicate pipettes (4-6 MΩ) manufactured by Narishige puller or pulled using NARISHIGE puller (NARISHIGE, PG10) from borosilicate tubing (Sutter Instruments) and filled by an internal solution containing (in mM): 140 CsMeS (CH3CsO3S), 5 KCl, 1 $MgCl_2$, 0.2 EGTA, 11 HEPES, 2 NaATP, 0.2 Na_2GTP pH 7.2–7.4 (pH 7.2**,** 290–295 mOsm).

The evoked postsynaptic inhibitory/excitatory responses of neurons in LV of PrL were elicited by field stimulation of excitatory and inhibitory afferents in LV. The low intensity pulses of stimulated current (25–100 μA, 100 μs duration) were applied through a fine-tipped (~2-3 μm), bipolar stimulating electrode made from borosilicate theta glass capillary tubing (Warner Instruments).

AMPA-receptor-mediated excitatory postsynaptic currents (EPSCs) were recorded in voltage clamp mode at -70 mV (reversal potential for GABA current) to minimize $GABA_A$ current. Inhibitory postsynaptic currents (IPSCs) mediated by $GABA_A$ receptors were recorded in voltage clamp mode at 0 mV (reversal potential for AMPA currents) to eliminate the current through AMPA-receptors. To calculate E/I (AMPA/GABA) ratio the responses, evoked by field stimulation of bipolar stimulating electrode, the amplitude of the AMPA response recorded at -70 mV was divided by the amplitude of the GABA response at 0 mV.

All recording data were acquired and analyzed by amplifier AXOPATCH 200B (Axon Instruments), digitizer BNC2090 (X National instruments) and software AxoGraph v.1.7.0,

Clampfit v.8.0 (pClamp, Molecular devices) and MiniAnalysis Program v.6.0.9 (Synaptosoft). Data were filtered at 2 kHz by AXOPATCH 200B amplifier (Axon Instruments) and digitized at 10-20 kHz via AxoGraph v.1.7.0. software.

RNA-sequencing

Tissue punches containing the rat PrL were extracted from virally infected tissue using a 2mm biopsy punch. Tissue was processed for RNA extraction using a modified manufacturer's procedure (miRNA Easy Kit, Qiagen). Briefly, RNA is extracted using phenol/chloroform, bound to the column, and washed with included buffers. During the wash steps, samples were treated for 15 mins at room temperature with RNase-free DNAse1 (Qiagen), followed by final washing and elution. RNA concentration and OD260/280 were determined by nanodrop. Frozen samples were sent to BGI Americas Cooperation (Cambridge, MA) and sequencing was performed using polyA mRNA directional RNA-seq library preparation using a DNBSEQ™ platform.

RNA-seq alignment and quality control

Quality of raw sequence data was assessed using FastQC (v0.11.9). Reads were aligned to the rat mRatBn7.2 reference genome using STAR (v2.7.1a)(5). Secondary alignment and multi-mapped reads were further removed using in-house bash scripts. Only uniquely mapped reads were retained for further analyses. Quality control metrics were assessed by Picard tool (http://broadinstitute.github.io/picard/). Ensembl gene annotation for mRatBn7.2 was used as reference alignment and downstream gene quantification. Gene level expression was calculated using featureCounts (v2.0.1)(6). Counts were calculated based on protein-coding genes from the annotation file.

Differential expression

Counts were normalized using counts per million reads (CPM). Genes with no reads were removed. To infer potential experimental confounders, we calculated surrogates variables were calculated using the sva package in R(7). Differential expression analysis was performed using a linear model post-hoc analysis in R using emmeans (v1.34)(8) with the following model: gene expression ~ Genotype * Condition. We estimated log2 fold changes and P-values. P-values were adjusted for multiple comparisons using TukeyHSD. Differentially expressed genes where consider for FDR<0.05 and a fold-change ≥ |0.3| as statistically significant. Rat Gene ID were translated into mouse and human Gene ID using biomaRt package in R(9).

Gene Ontology (GO) analysis

The functional annotation of differentially expressed and co-expressed genes was performed using clusterProfiler (v4.2) and enrichR (v3.2). A Benjamini-Hochberg FDR (FDR<0.05) was applied as a multiple comparison adjustment.

Code availability
Codes to support the DGE analysis: https://github.com/BertoLabMUSC/Barry_HDAC5.

Availability of data and material.
The NCBI GEO accession number for the data reported in this manuscript is **GSE277932**.

Supplemental References

1. Li X, Carreria MB, Witonsky KR, Zeric T, Lofaro OM, Bossert JM, et al. (2018): Role of Dorsal Striatum Histone Deacetylase 5 in Incubation of Methamphetamine Craving. *Biological psychiatry*.1-10.
2. Anderson EM, Tsvetkov E, Galante A, DeVries D, McCue LM, Wood D, et al. (2023): Epigenetic function during heroin self-administration controls future relapse-associated behavior in a cell type-specific manner. *Proc Natl Acad Sci U S A*. 120:e2210953120.
3. Taniguchi M, Carreira MB, Smith LN, Zirlin BC, Neve RL, Cowan CW (2012): Histone deacetylase 5 limits cocaine reward through cAMP-induced nuclear import. *Neuron*. 73:108-120.
4. Barry SM, McGinty JF (2017): Role of Src Family Kinases in BDNF-Mediated Suppression of Cocaine-Seeking and Prevention of Cocaine-Induced ERK, GluN2A, and GluN2B Dephosphorylation in the Prelimbic Cortex. 42:1972-1980.
5. Dobin A, Davis CA, Schlesinger F, Drenkow J, Zaleski C, Jha S, et al. (2013): STAR: ultrafast universal RNA-seq aligner. *Bioinformatics*. 29:15-21.
6. Liao Y, Smyth GK, Shi W (2014): featureCounts: an efficient general purpose program for assigning sequence reads to genomic features. *Bioinformatics*. 30:923-930.
7. Leek JT, Johnson WE, Parker HS, Jaffe AE, Storey JD (2012): The sva package for removing batch effects and other unwanted variation in high-throughput experiments. *Bioinformatics*. 28:882-883.
8. Searle SR, Speed FM, Milliken GA (1980): Population Marginal Means in the Linear Model: An Alternative to Least Squares Means. *The American Statistician*. 34:216-221.
9. Smedley D, Haider S, Ballester B, Holland R, London D, Thorisson G, et al. (2009): BioMart – biological queries made easy. *BMC Genomics*. 10:22.
